# Supplementary material for: In-fibre logic and memory via tuneable passivation–corrosion
Source: Nat Commun. 2026 Mar 31;17:4666. doi: 10.1038/s41467-026-71249-7 (PMC13201798; doi:10.1038/s41467-026-71249-7)
Supplement: Supplementary file 1 — Supplementary Information [file 41467_2026_71249_MOESM1_ESM.pdf]

Supplementary Information for

In-fibre logic and memory via tunable passivation–corrosion

Yuanlong Li [1†], Weifeng Yang [1†]*, Alexander V. Shokurov [1], Manuel Reis Carneiro [1], Carlo Menon[1]*

1 Biomedical and Mobile Health Technology Laboratory, Department of Health Sciences and Technology, ETH Zurich, Lengghalde 5, 8008 Zurich, Switzerland.

†These authors contributed equally to this work.

*Corresponding author: Weifeng Yang (weifeng.yang@hest.ethz.ch), Carlo Menon (carlo.menon@hest.ethz.ch)

Supplementary Figures

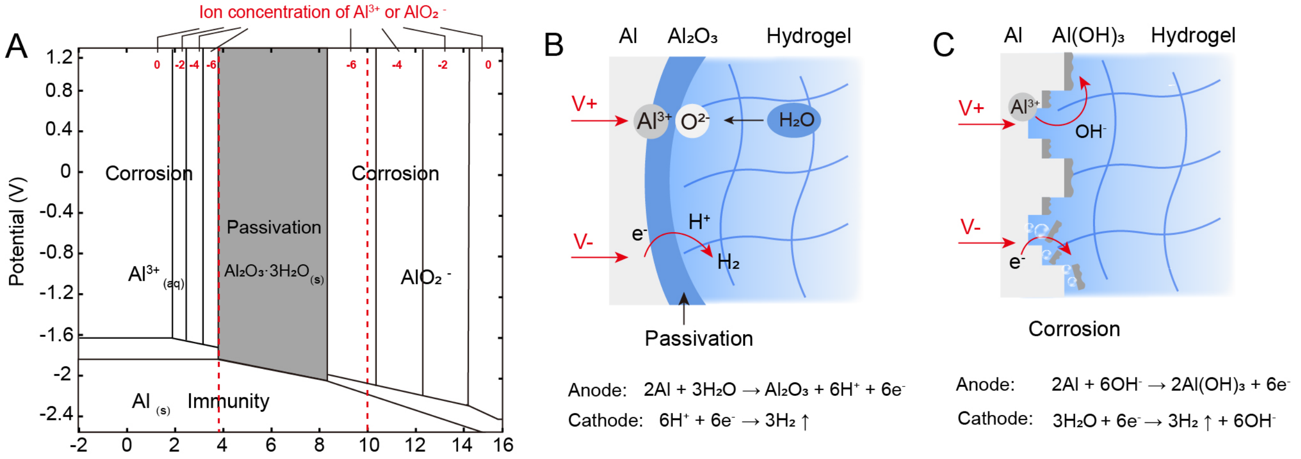

Supplementary Figure 1. Passivation and corrosion effects on aluminum electrode interface. A. Potential-pH electrochemistry diagram for aluminum [1], [2]. **B.** The passivation reaction equation of the aluminum electrode interface under weak acid conditions. **C.** Corrosion reaction equation of aluminum electrode interface under weak alkaline conditions.

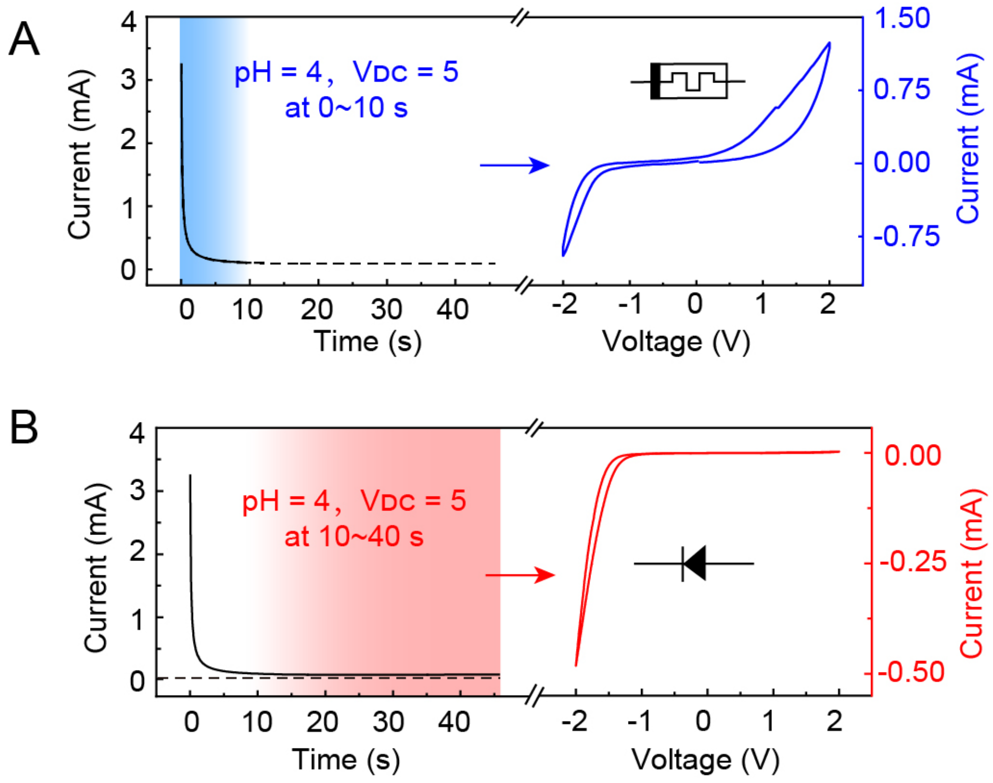

Supplementary Figure 2. Pre-applying voltage and time to control FLAME to behave as a diode/memristor device under weak acidic conditions. A. Short-term positive voltage treatment partially passivates the aluminum interface and behaves as a memristor. **B.** Long-term positive voltage treatment makes the aluminum interface completely passivated and behaves as a diode.

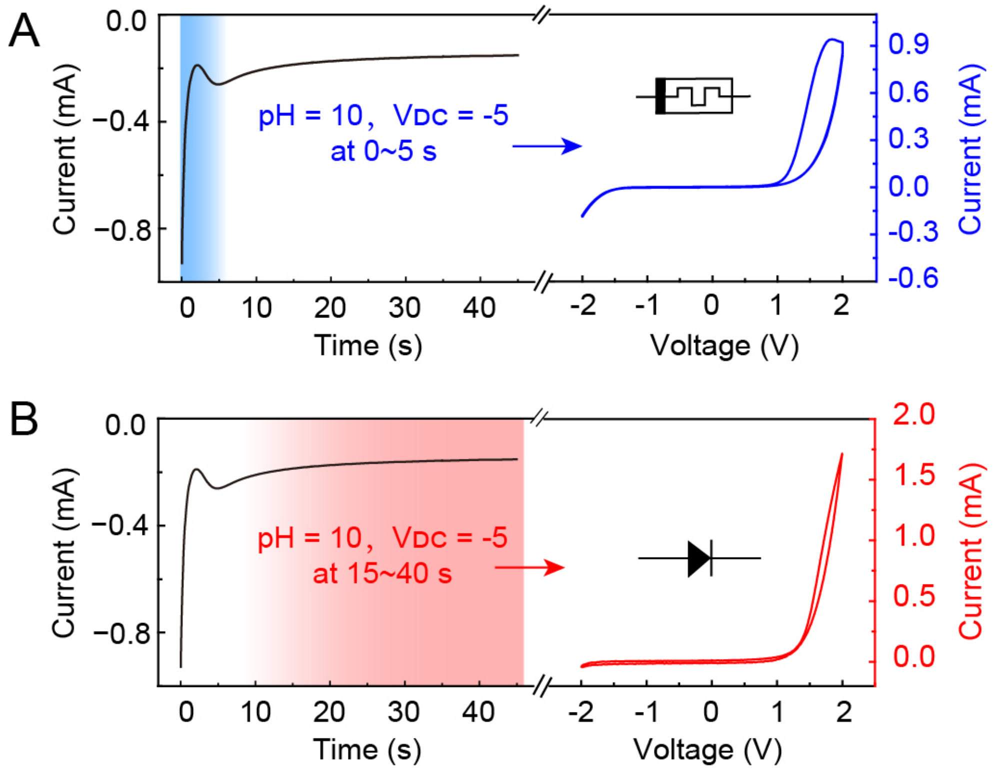

Supplementary Figure 3. Pre-applying voltage and time to control FLAME to behave as a diode/memristor device under weak alkaline conditions. A. Short-term negative voltage treatment causes partial corrosion of the aluminum interface and manifests as a memristor. **B.** Long-term negative voltage treatment causes a local high hydroxide concentration at the aluminum interface, making it behave as a diode.

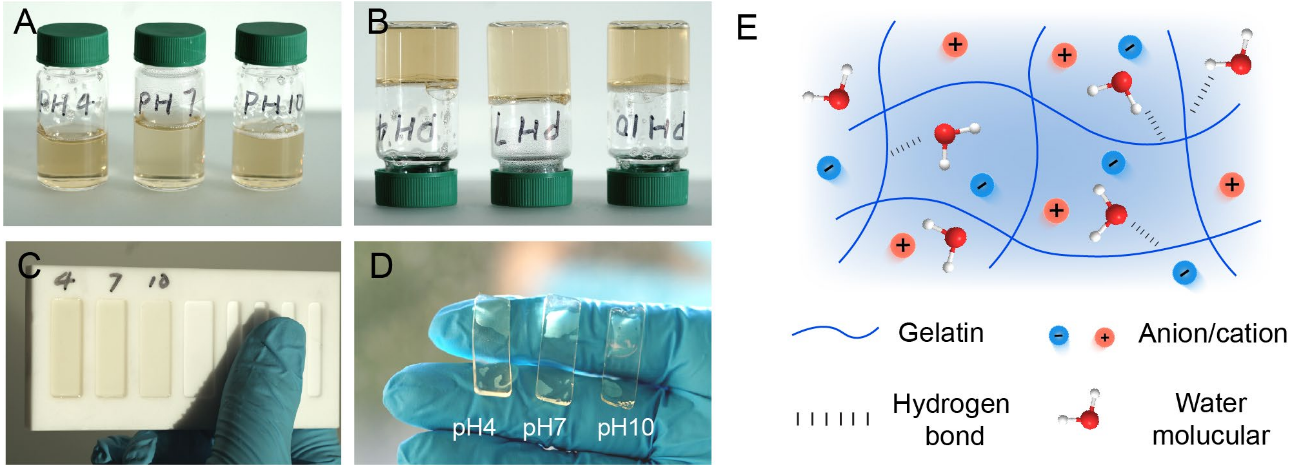

Supplementary Figure 4. Gelatin-based hydrogels prepared in pH 4, 7, and 10 buffers. A-D. Self-supporting hydrogels can be prepared in the gelatin system in buffer solutions of pH 4, 7, and 10. **E.** Molecular chain network of gelatin hydrogel.

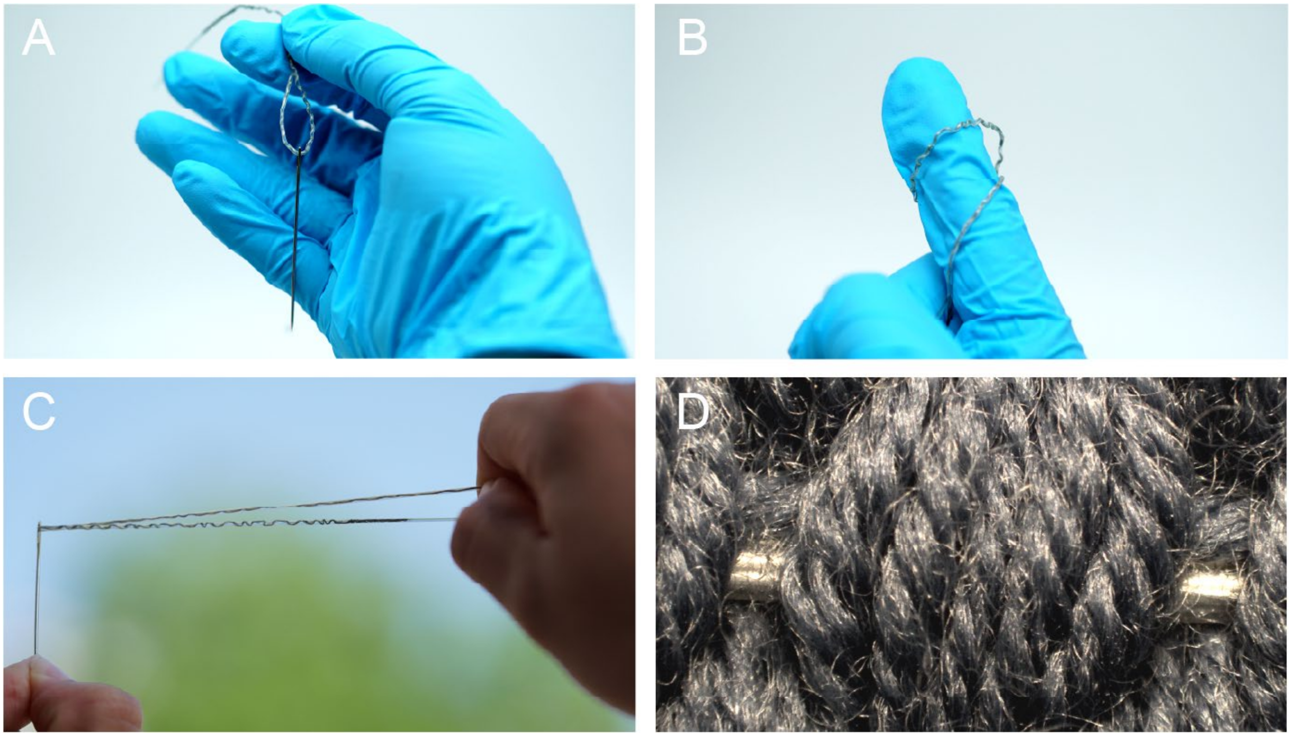

Supplementary Figure 5. FLAME with built-in helical structure based on gelatin hydrogel and aluminum yarn. A. FLAME can be inserted into the needle smoothly. **B.** FLAME can be wrapped around fingers at multiple angles. **C.** Digital photo of FLAME stretched after being threaded. **D.** Digital photo of woven textile made of FLAME and cotton yarn.

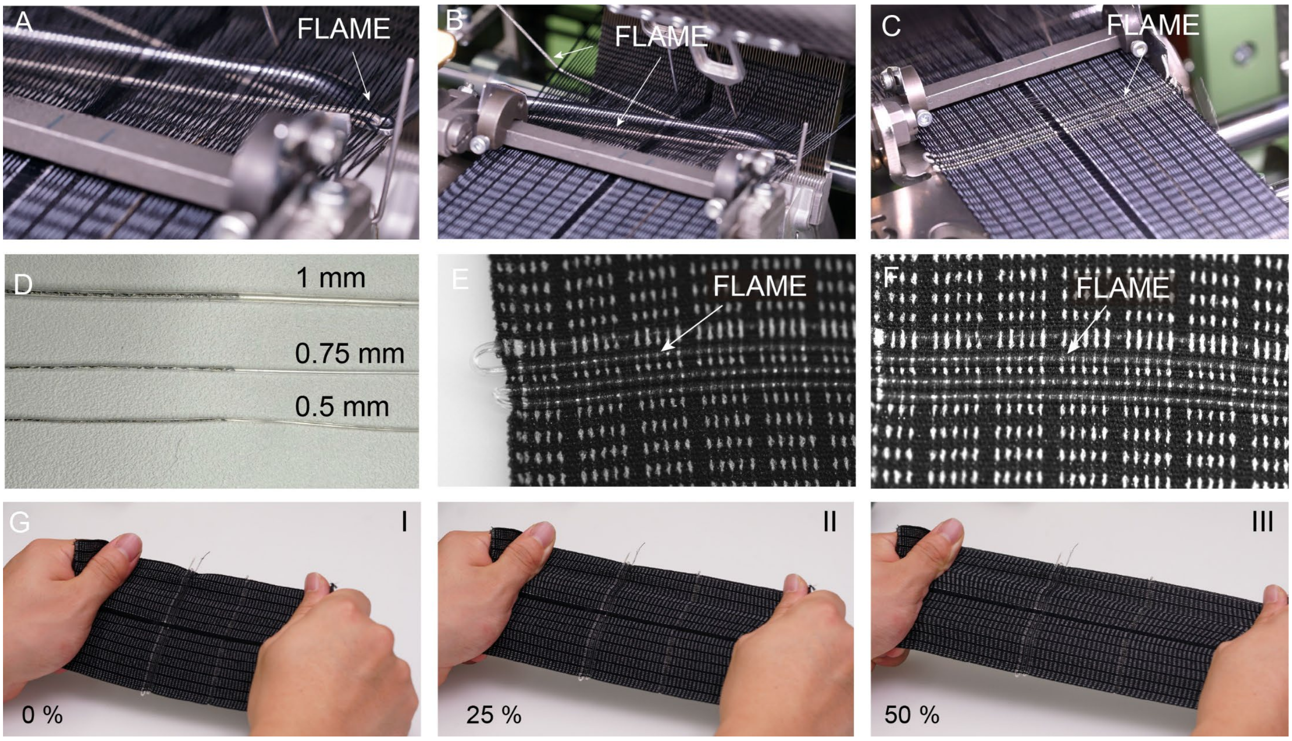

Supplementary Figure 6. Industrial-scale integration and mechanical characterization of FLAME fibres. (A–C) Digital photographs showing the integration of FLAME fibres into textiles using a commercial industrial weaving machine. **(D)** Photographs of FLAME fibres with various diameters (0.5 mm, 0.75 mm, and 1.0 mm). **(E, F)** Magnified views of the woven electronic textile, illustrating the seamless integration of FLAME as functional yarns within the fabric structure. **(G)** Photographs of the electronic textile under different tensile strains (0%, 25%, and 50%), showcasing its excellent structural integrity and mechanical stretchability for wearable applications.

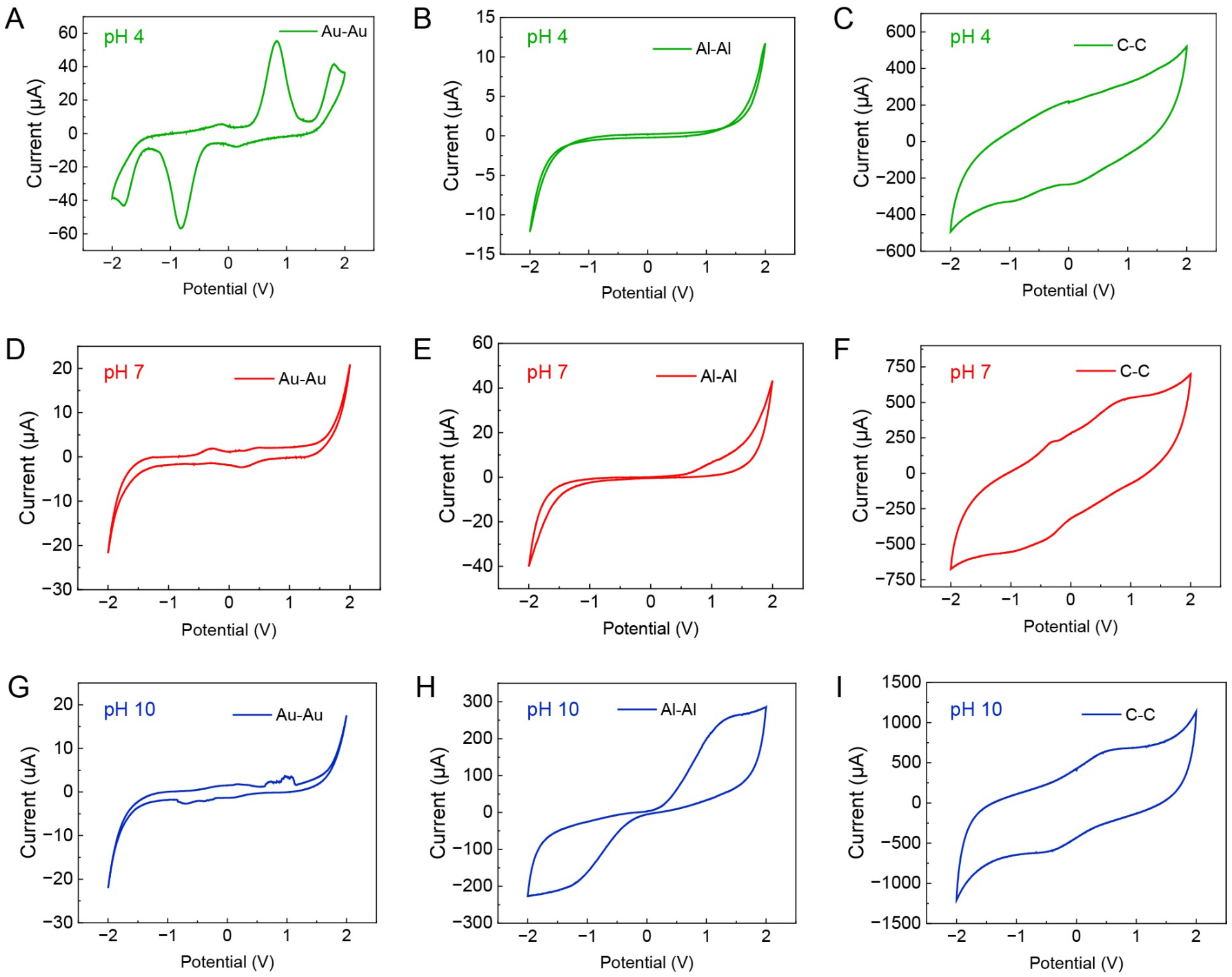

Supplementary Figure 7. IV curves under different electrode systems and different pH buffer solutions. IV curves of **A.** gold-gold electrode, **B.** aluminum-aluminum electrode, and **C.** carbon-carbon electrode in pH 4 buffer solution. IV curves of **D.** gold-gold electrode, **E.** aluminum-aluminum electrode, and **F.** carbon-carbon electrode in pH 7 buffer solution. IV curves of **G.** gold-gold electrode, **H.** aluminum-aluminum electrode, and **I.** carbon-carbon electrode in pH 7 buffer solution.

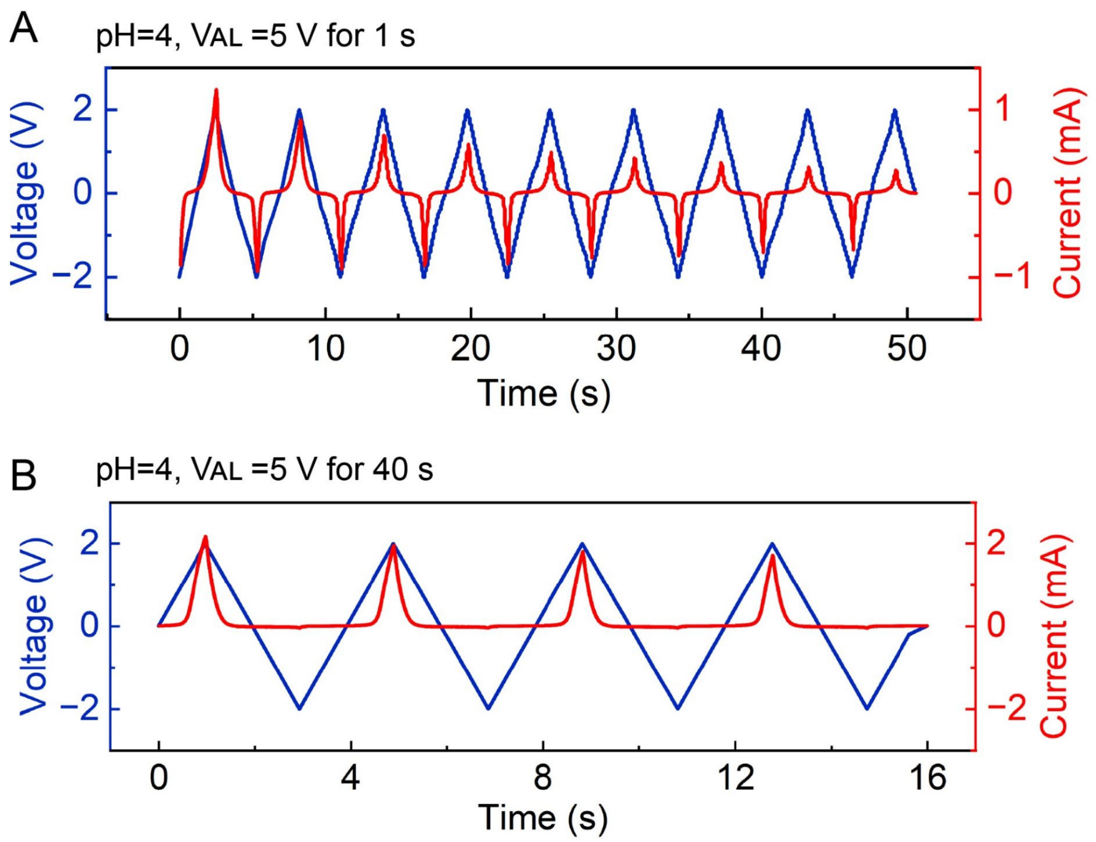

Supplementary Figure 8. IV curve of FLAME under weak acidic conditions. A. At pH = 4, the aluminum side was pretreated with a 5V voltage for 1 second, **B.** At pH = 4, the aluminum side was pretreated with a 5V voltage for 20 seconds, and then a -2~2V voltage was applied to the time-voltage/current curve under cyclic scanning.

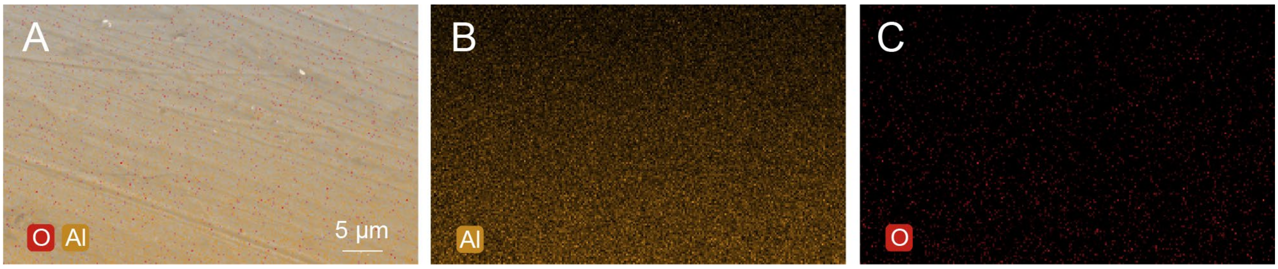

Supplementary Figure 9. SEM mapping for pristine aluminum. A. Mixed mapping of Al and O in pristine aluminum. **B.** Mapping of Al element of pristine aluminum. **C.** Mapping of O element of pristine aluminum.

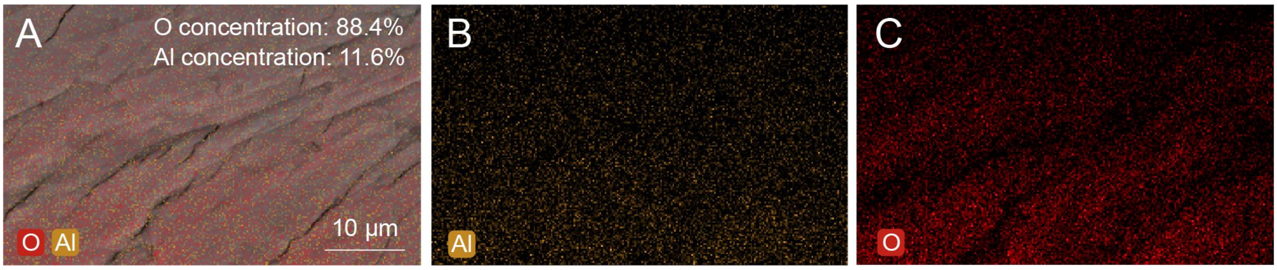

Supplementary Figure 10. SEM and mapping photos of the aluminum side after 5 V positive voltage for 40 s at pH = 4 buffer. A. Mixed mapping of Al and O for sample at 5 V positive voltage for 40s. **B.** Mapping of Al element for sample at 5 V positive voltage for 40s. **C.** Mapping of O element for sample at 5 V positive voltage for 40 s.

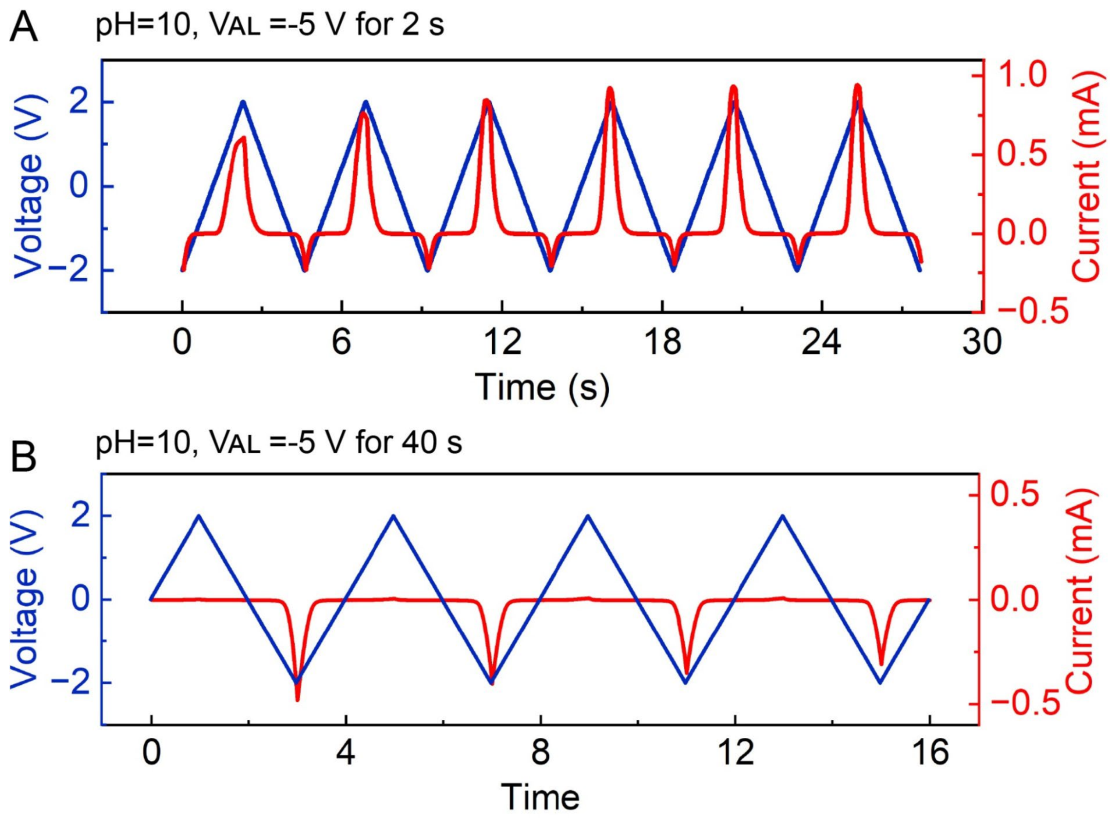

Supplementary Figure 11. IV curve of FLAME under weak alkaline conditions. A. At pH = 10, the aluminum side was pretreated with a -5 V voltage for 2 s, **B.** At pH = 10, the aluminum side was pretreated with a -5 V voltage for 40 seconds, and then a -2~2V voltage was applied to the time-voltage/current curve under cyclic scanning.

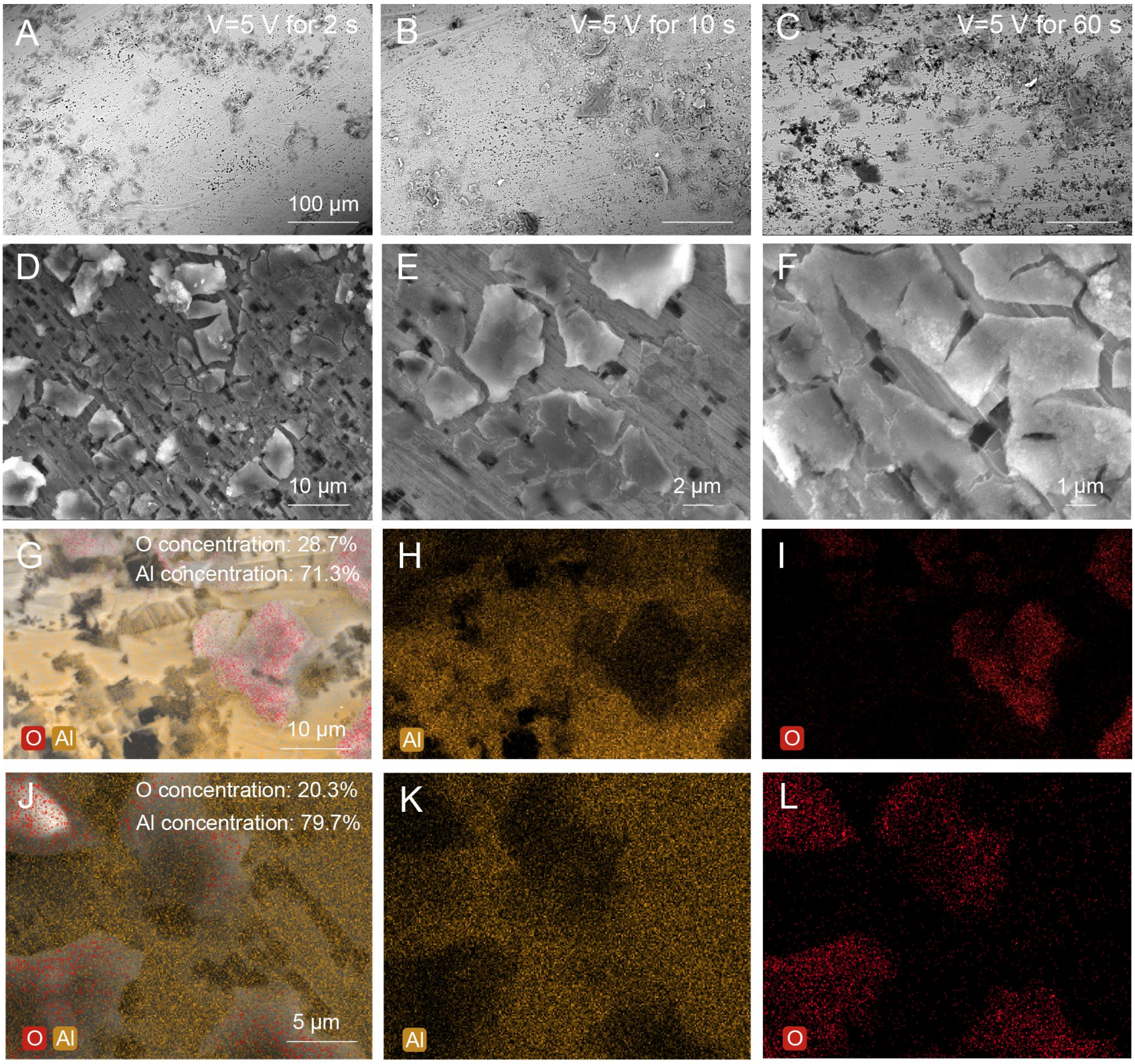

Supplementary Figure 12. SEM and mapping photos of the aluminum side after 5 V voltage treatment at pH= 10 buffer. A-C. SEM images of the aluminum side under positive pressure for 2 s, 10 s and 60 s in pH=10 buffer. **D-F.** SEM images at different magnifications after positive voltage on the aluminum side for 10 seconds in pH=10 buffer. **G-L.** Mapping image of aluminum surface after 5 V voltage treatment at pH= 10 buffer.

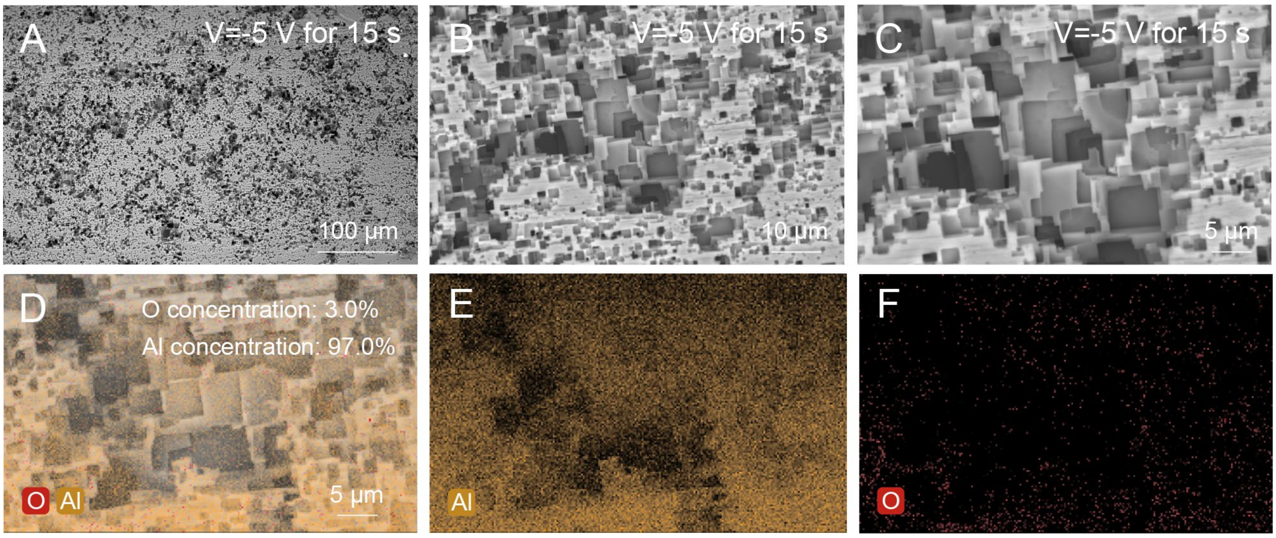

Supplementary Figure 13. SEM and mapping photos of the aluminum side after 5 V and reverse -5 V treatment at pH= 10 buffer. A-C. SEM and **D-F.** mapping images of aluminum surface.

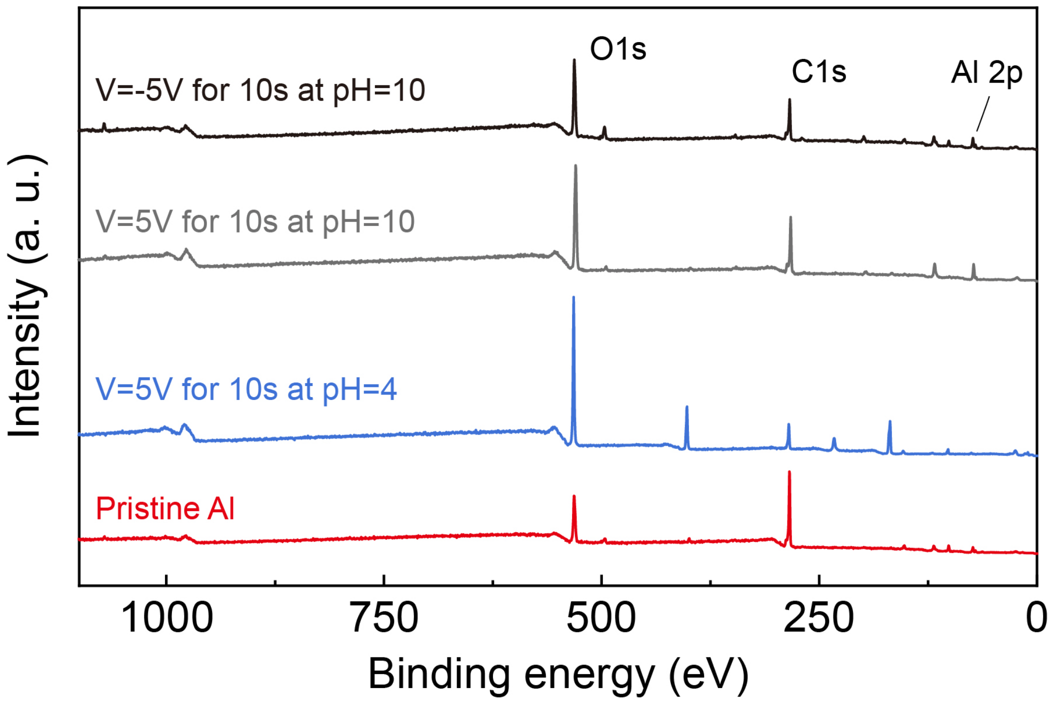

Supplementary Figure 14. X-ray photoelectron spectroscopy (XPS) spectra for the surface of aluminum electrode.

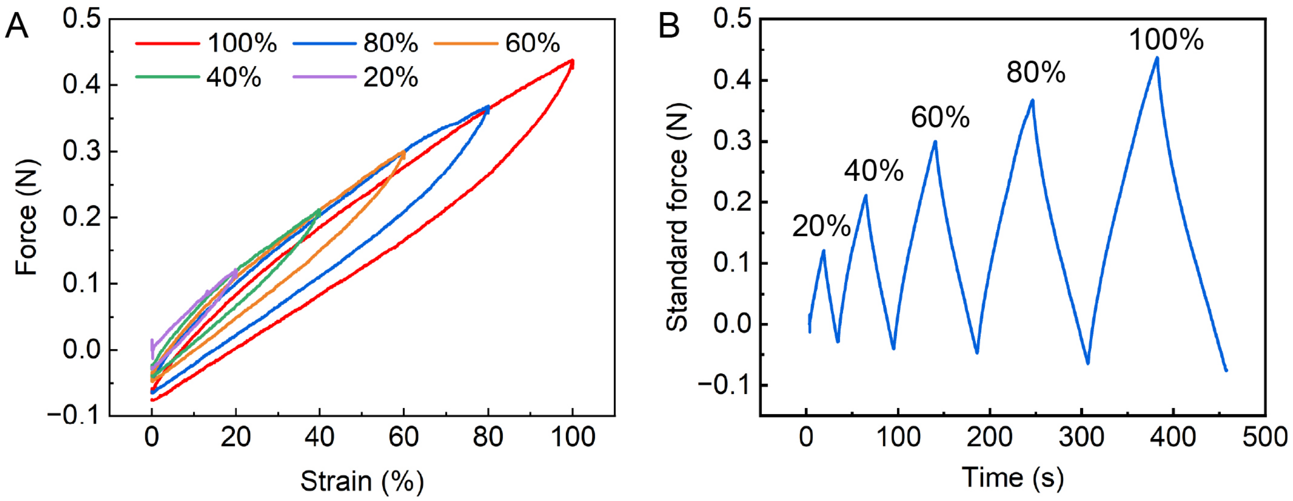

Supplementary Figure 15. Mechanical properties of textile integrated diodes. A. Sequential multistep stress−strain cycles of textile integrated diodes without timespans. **B.** Sequential multistep stress−time cycles of textile integrated diodes without timespans.

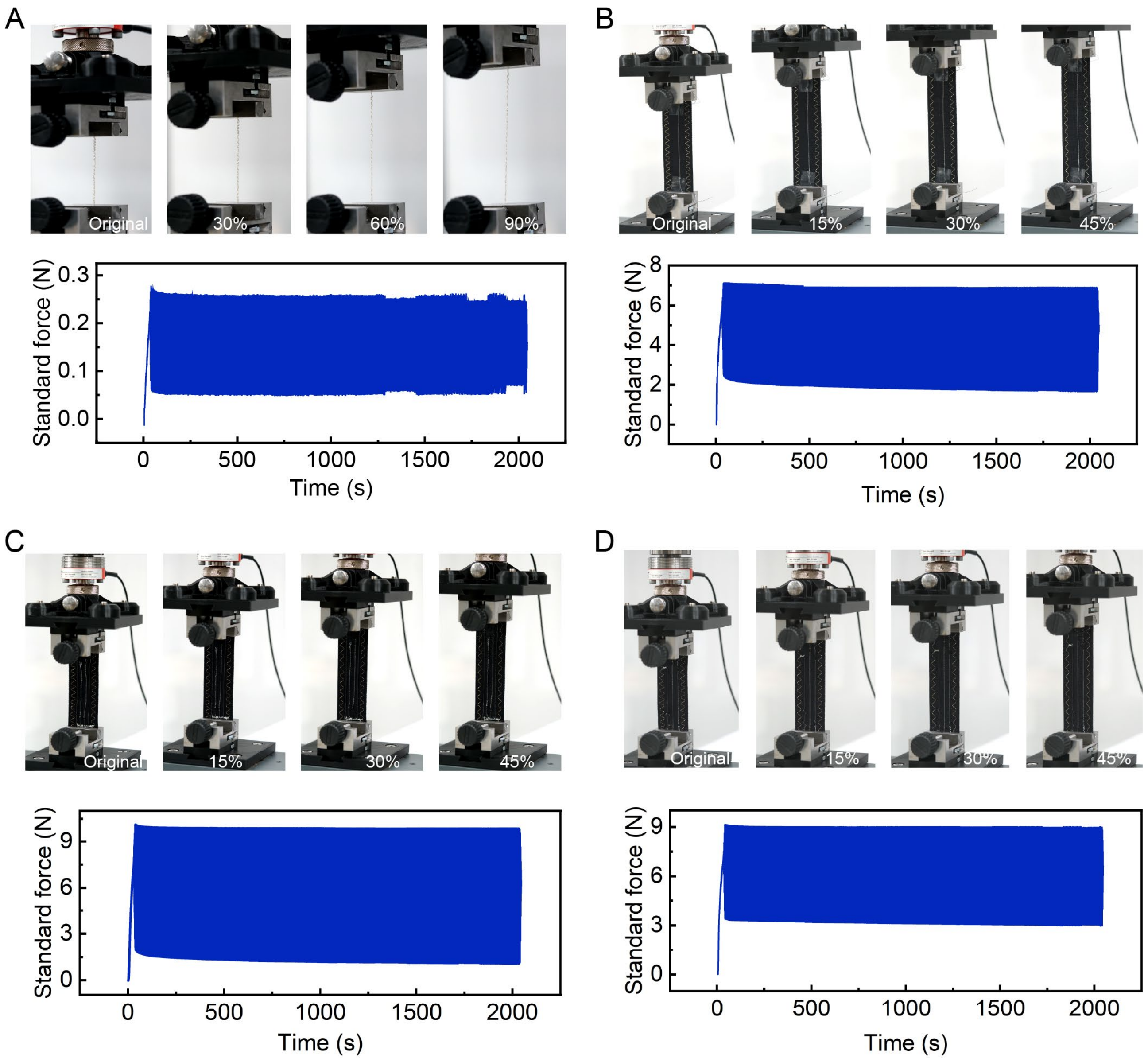

Supplementary Figure 16. Mechanical properties of FLAME and its logic gate textile. A. Stress-time cycle curve of FLAME under 0~90% strain. **B.** Stress-time cycle curve of FLAME integrated diode textile. **C.** "AND" logic gate textile and **D.** "OR" logic gate textile under 0~45% strain.

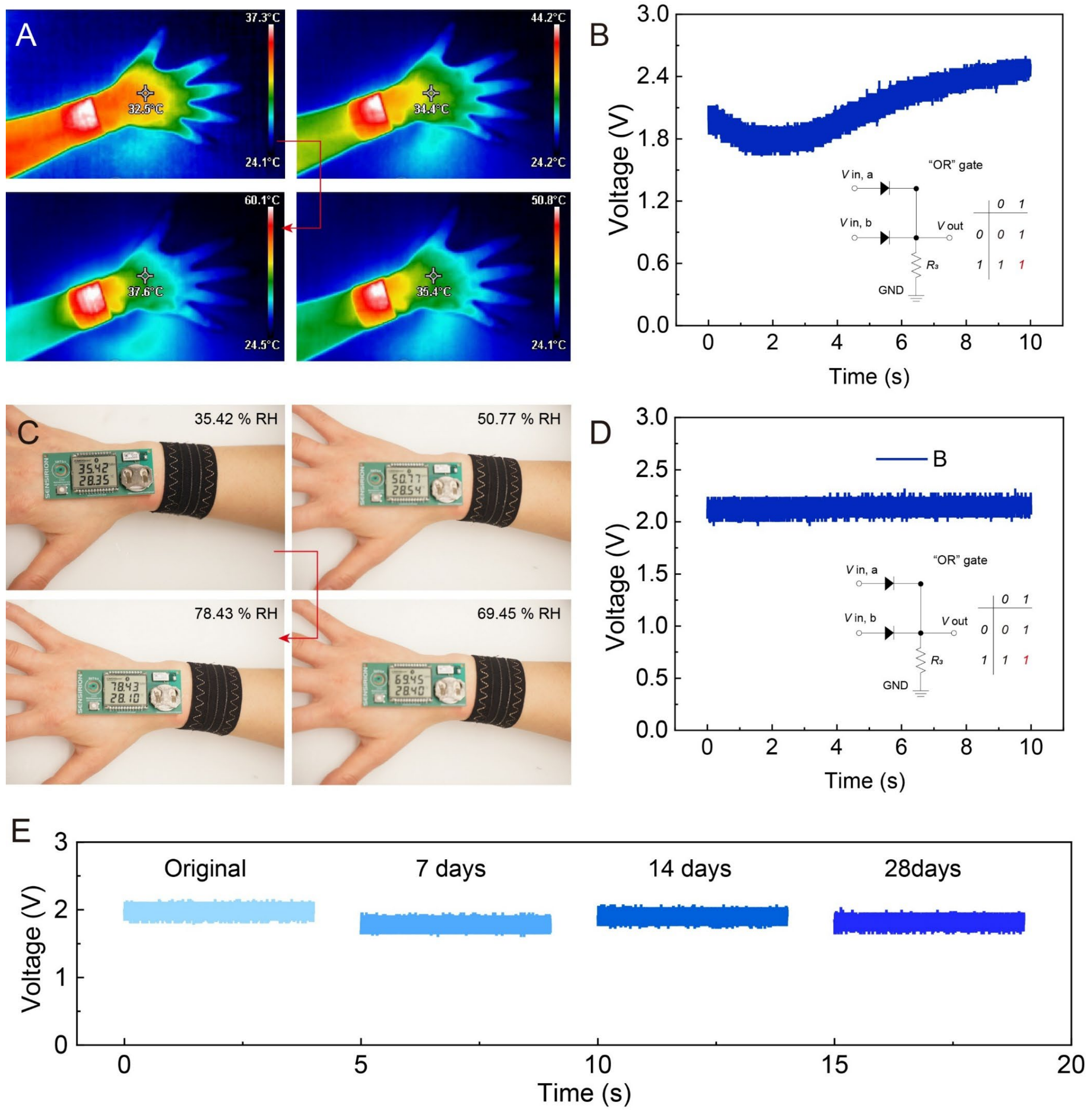

Supplementary Figure 17. Environmental durability and long-term stability characterization of the FLAME device. (A) Infrared thermography of the wearable logic gate wristband under heating, showing the skin surface temperature increasing from 32.5 °C to 60.1 °C. **(B)** Output voltage of the OR logic gate during the heating process; the high-level state remains stable despite a slight voltage drift. **(C)** Photographs of the device under varying micro-environmental humidity levels (from 35.42% RH to 78.43% RH) controlled by a humidifier and monitored by a sensor. **(D)** Corresponding output voltage of the OR logic gate at (1, 1) input, demonstrating excellent humidity resistance due to the protective silicone encapsulation. **(E)** Long-term stability test of the device over a 28-day storage period (Initial, 7 days, 14 days, and 28 days), confirming the structural and functional reliability.

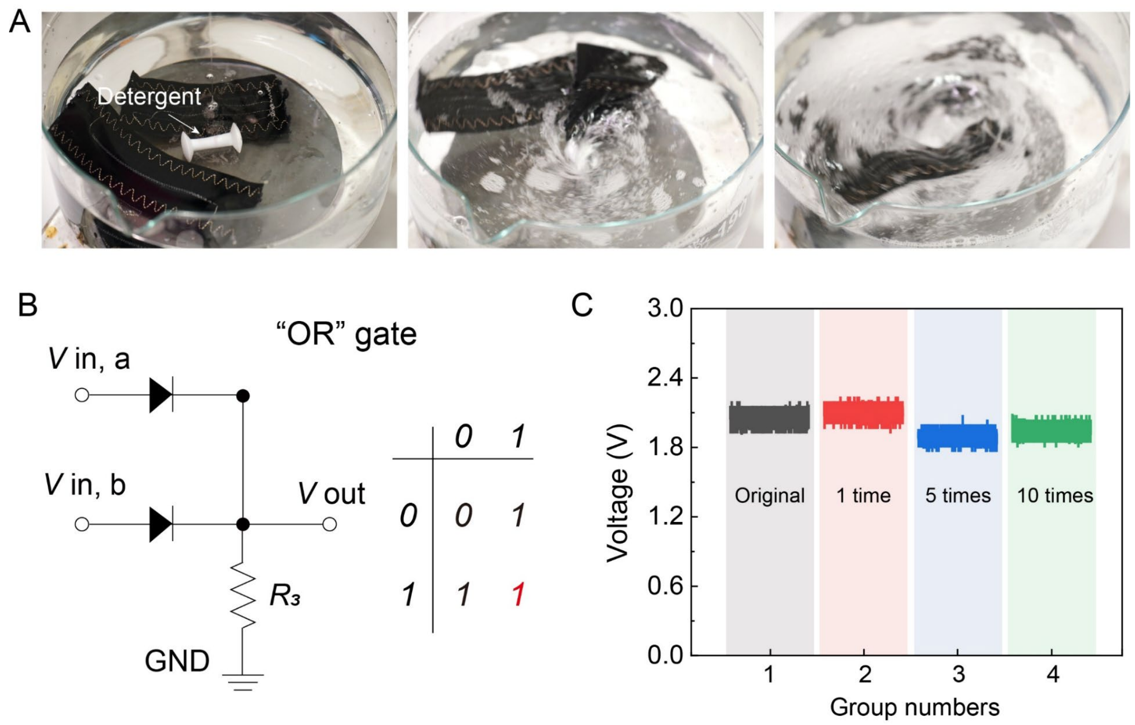

Supplementary Figure 18. Characterization of the washability and electrical stability of the FLAME textile. (A) Schematic and digital photographs of the simulated washing process. **(B, C)** Output voltage signals of the FLAME-based OR logic gate after 0, 1, 5, and 10 washing cycles.

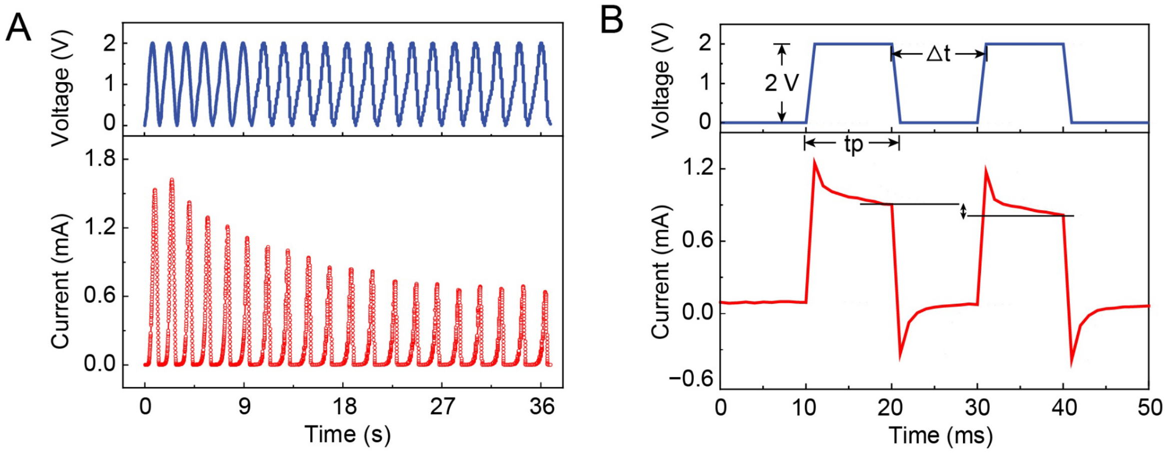

Supplementary Figure 19. Memristor performance of FLAME under weak acid (pH=4) conditions. A. Evolution of the current response under voltage pulses of constant polarity. **B.** STP test in fibre based memristor. The upper plots show voltage pulse waveforms.

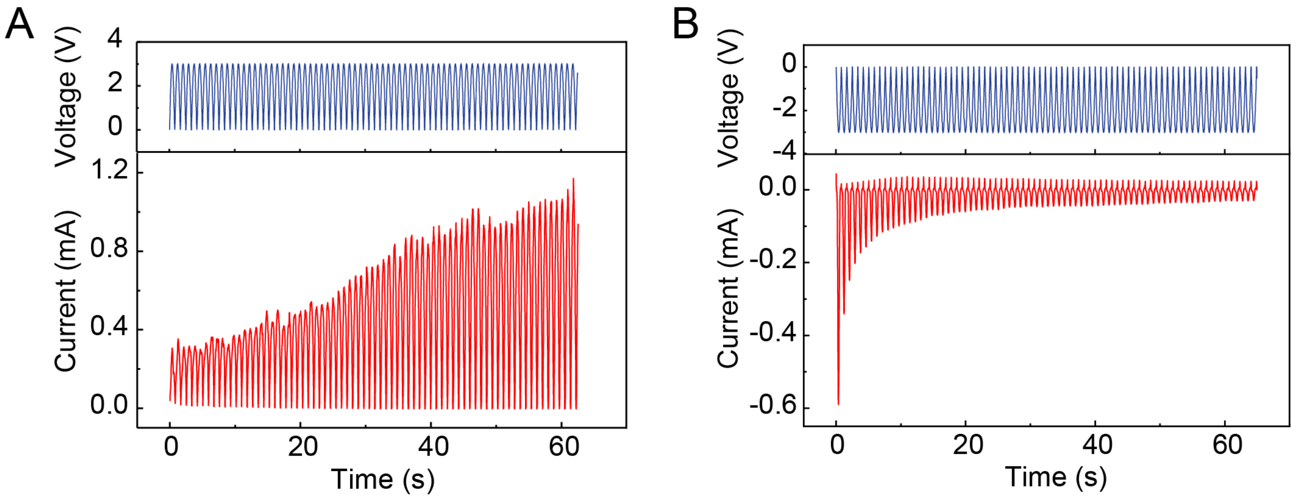

Supplementary Figure 20. Memristor performance of FLAME under weak alkaline (pH=10) conditions. Evolution of the current response under voltage pulses of constant polarity. **A.** Successive positive pulses result in an increase in conductance. **B.** Successive negative pulses result in a decrease in conductance.

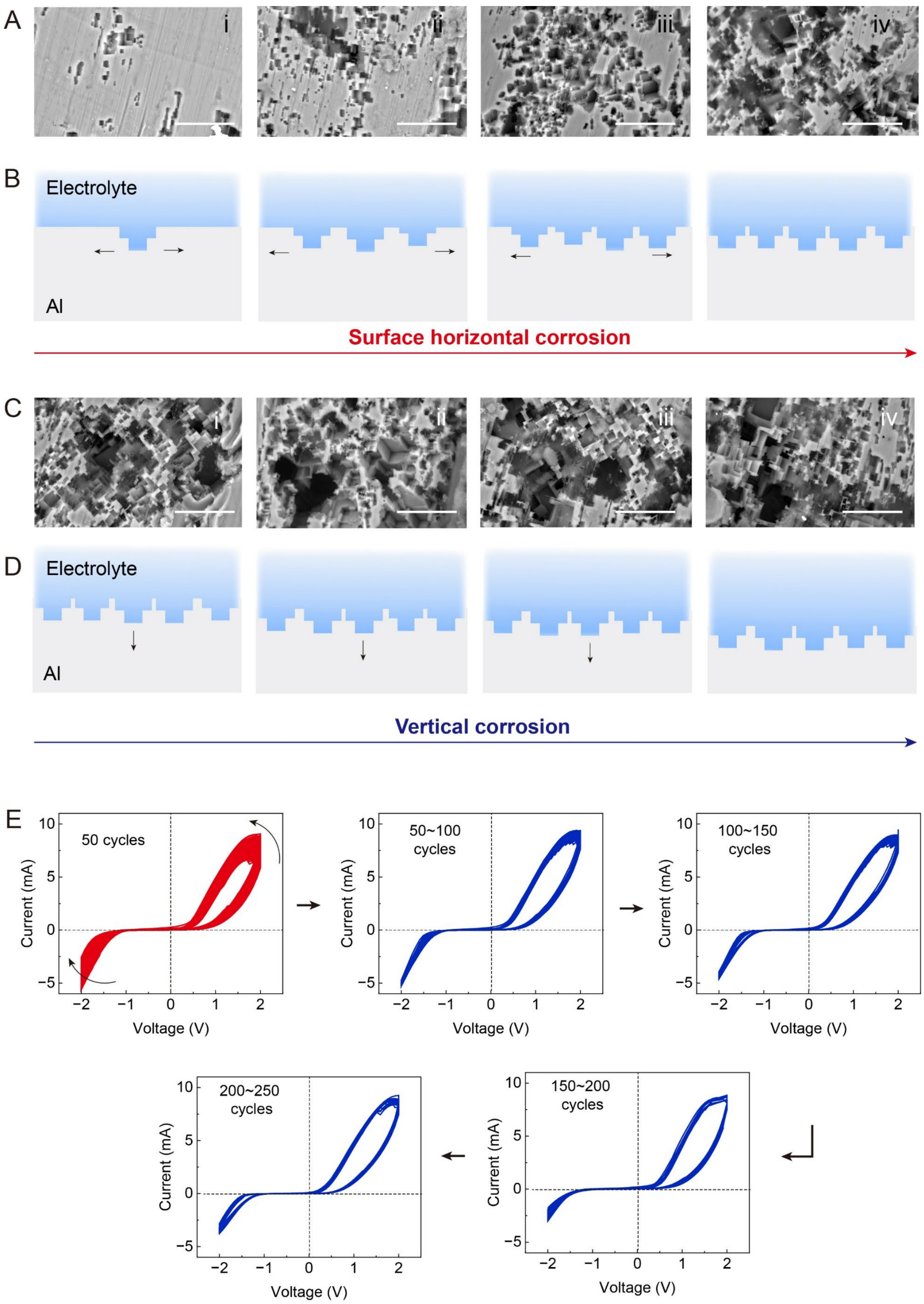

Supplementary Figure 21. Morphological evolution and electrochemical stability of the Al electrode under alkaline operation (pH=10). (A, B) SEM images and schematic illustration of the surface horizontal corrosion stage (initial 50 cycles), showing the formation and expansion of square pitting on the Al surface. **(C, D)** SEM images and schematic illustration of the vertical corrosion stage (50–250 cycles), where the corrosion propagates into the bulk while maintaining a stable active surface area. **(E)** Corresponding I-V curves of the FLAME memristor over 250 cycles.

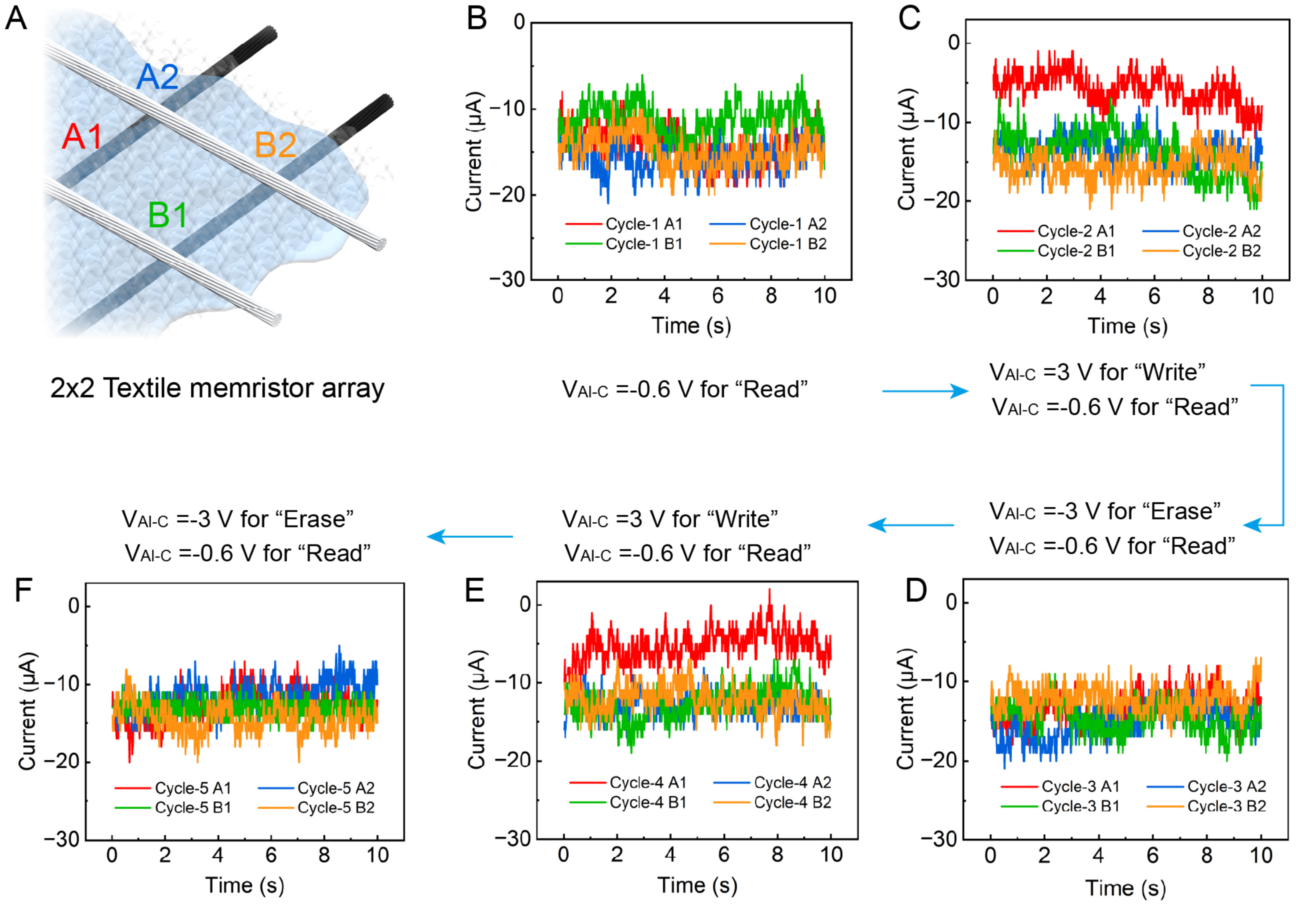

Supplementary Figure 22. Multiple "write" and "erase" performance of 2*2 textile memristor array. A. Schematic diagram of 2*2 textile memristor array. **B.** V_{AL-C}=-0.6V for "Read". **C.** V_{Al-C} =3 V for "Write" and V_{Al-C} =-0.6 V for "Read". **D.** V_{Al-C} =-3 V for "Erase" and V_{Al-C} =-0.6 V for "Read". **E.** V_{Al-C} =3 V for "Write" and V_{Al-C} =-0.6 V for "Read". **F.** V_{Al-C} =-3 V for "Erase" and V_{Al-C} =-0.6 V for "Read".

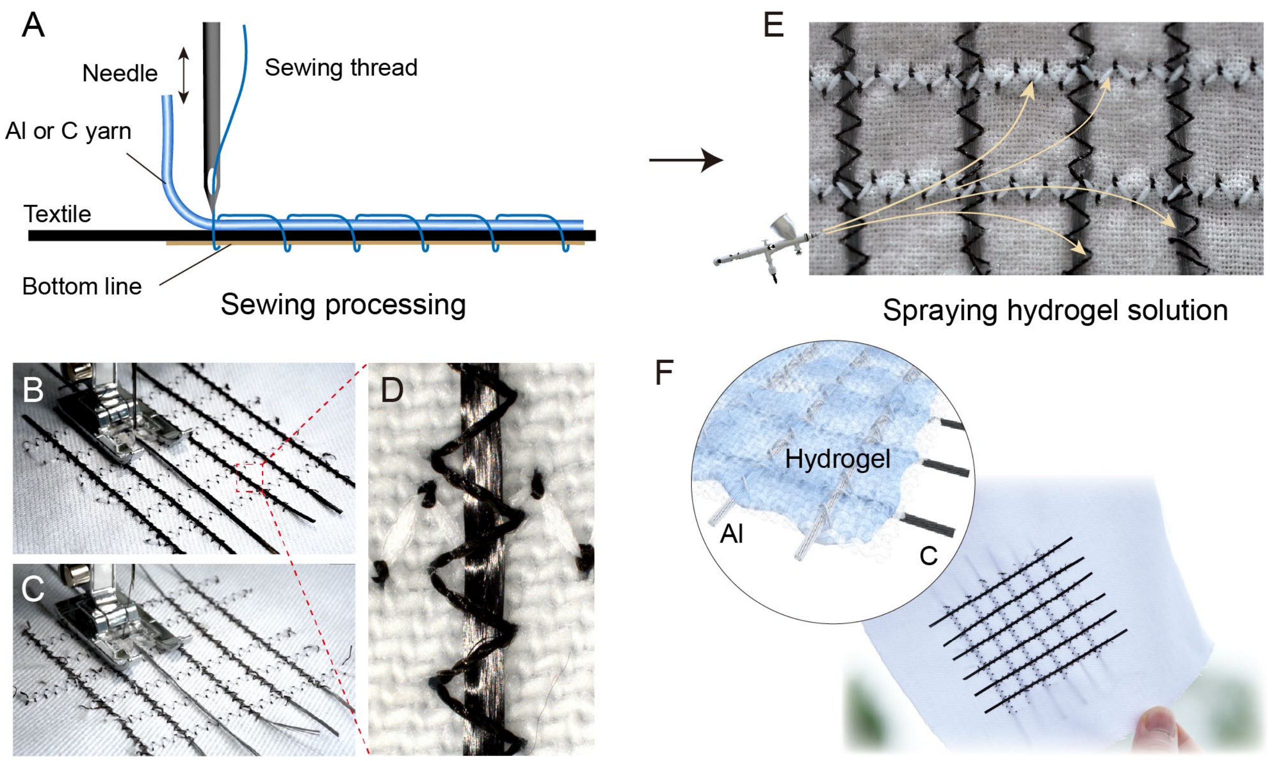

Supplementary Figure 23. Fabrication process of 6*6 textile memristor array. A. Sewing process diagram for carbon and aluminum yarn on commercial textiles. **B.** Sewing carbon fibre on top side of hydrophilic cotton textile. **C.** Sewing aluminum yarn on bottom-side of hydrophilic cotton fabric. **D.** Detailed enlargement of the sewing process. **F.** Schematic diagram and digital photo of 6*6 textile memristor array.

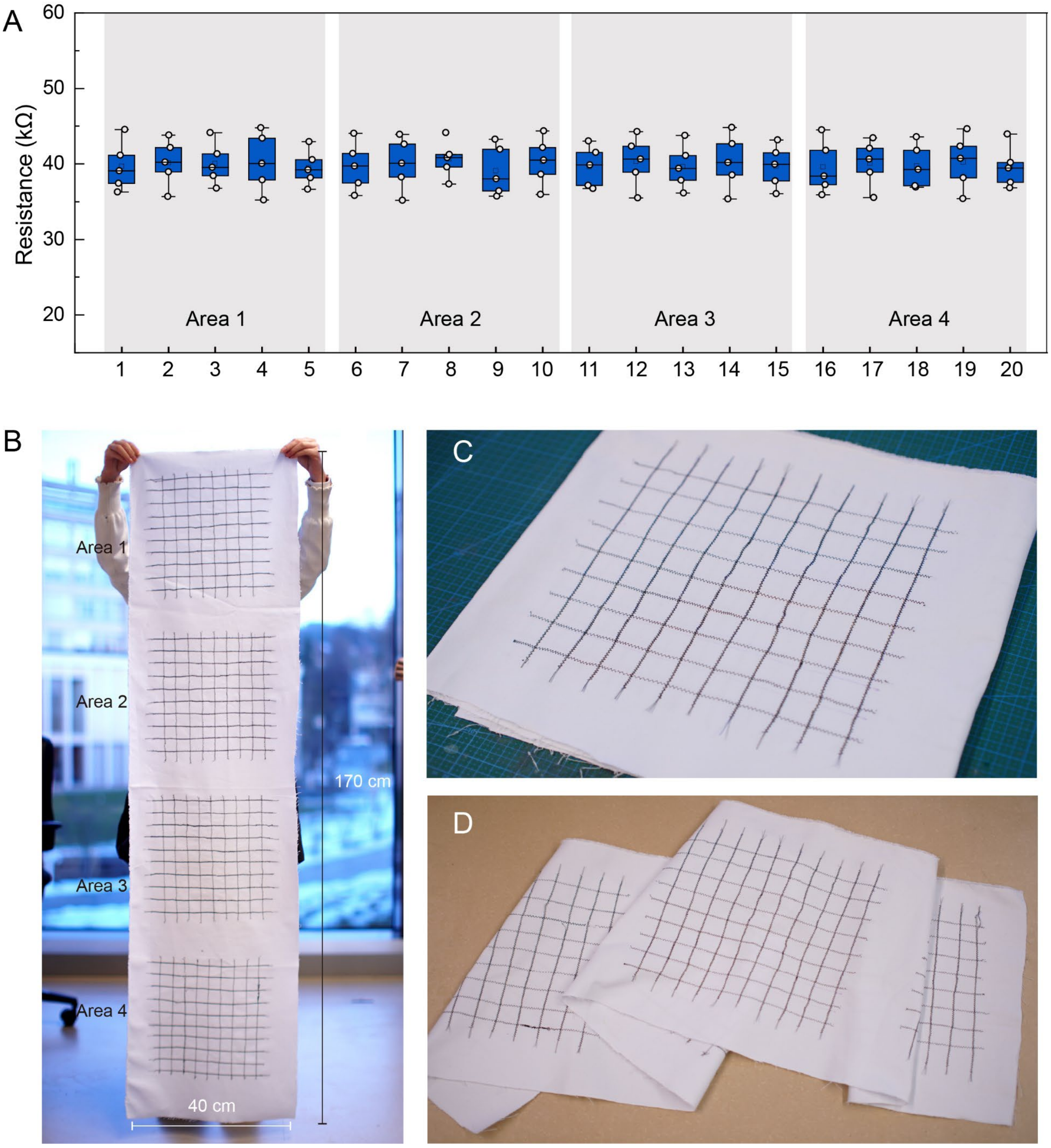

Supplementary Figure 24. Characterisation of device-to-device variation in a large-area memristor textile array. (A) Statistical box plot of the initial resistance values for 20 randomly selected memristor units across four different functional areas. **(B-D)** Digital photographs of the large-scale integrated textile (approx. 40 cm * 170 cm) containing multiple memristor logic areas, showcasing the reliability of the FLAME fabrication process.

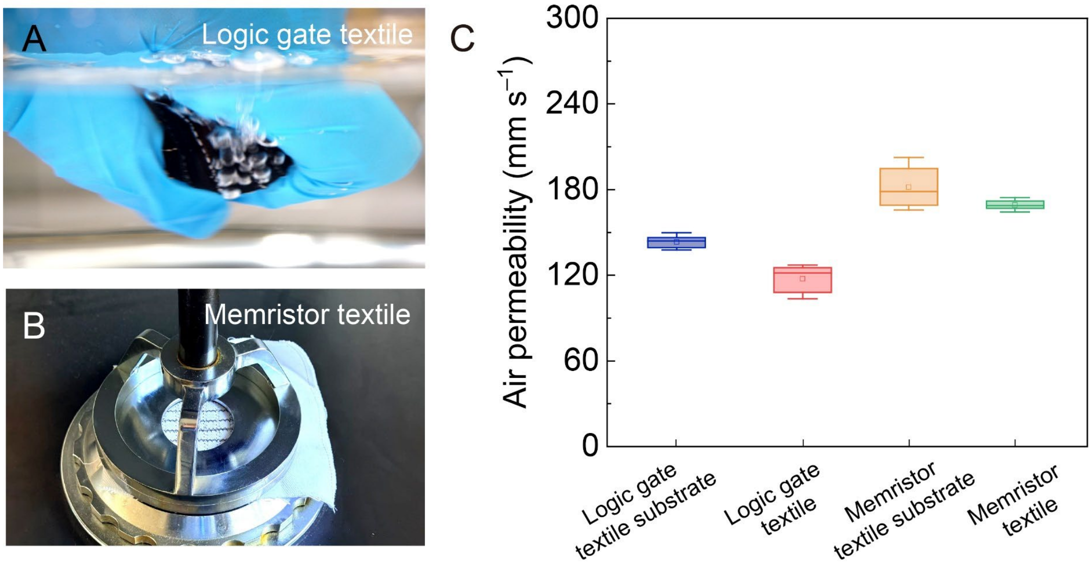

Supplementary Figure 25. Characterization of the breathability of the FLAME device. (A) Digital photograph of the qualitative breathability test, where air bubbles successfully pass through the submerged electronic textile. **(B)** Experimental setup of the air permeability tester used for quantitative measurement. **(C)** Comparison of air permeability between the original textile substrates and the integrated electronic textiles at a pressure of 100 Pa.

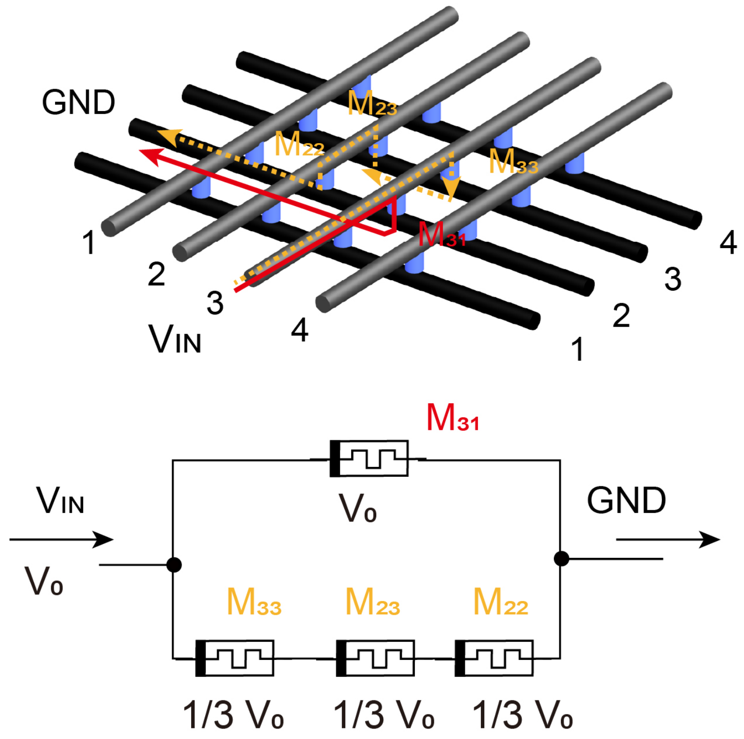

Supplementary Figure 26.Analysis of crosstalk suppression in the memristor array. Schematic of the crossbar array showing the addressing of device M_{31} and the corresponding equivalent circuit for the sneak path involving adjacent devices M_{33}, M_{23} and M_{22}.

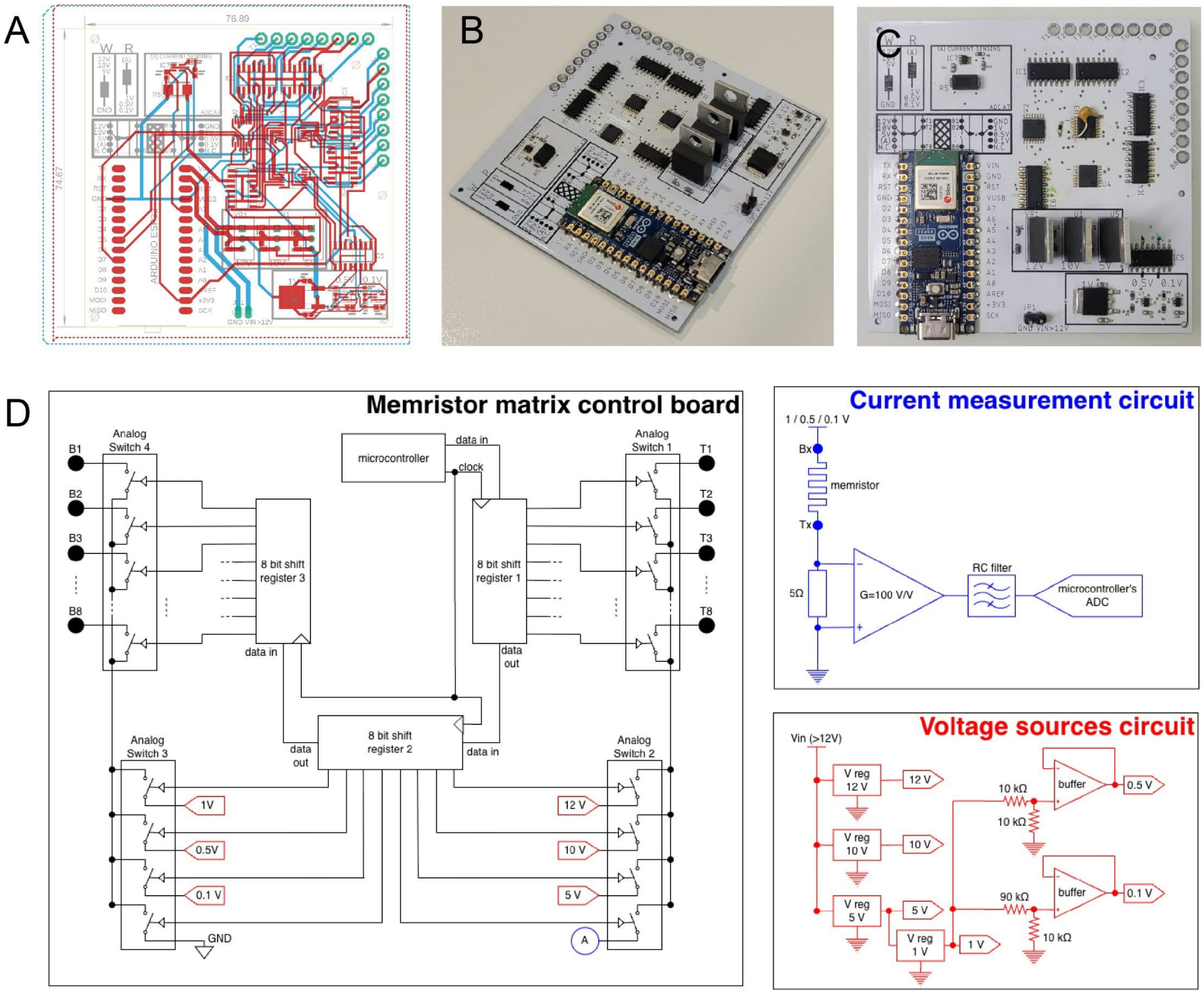

Supplementary Figure 27. Portable control and signal acquisition system for the memristor array. (A-C) PCB layout and photograph of the custom-designed memristor matrix control board. **(D)** Detailed circuit schematics, including the 8×8 switch matrix architecture, the current measurement circuit (amplification and filtering), and the multi-level voltage source circuit.

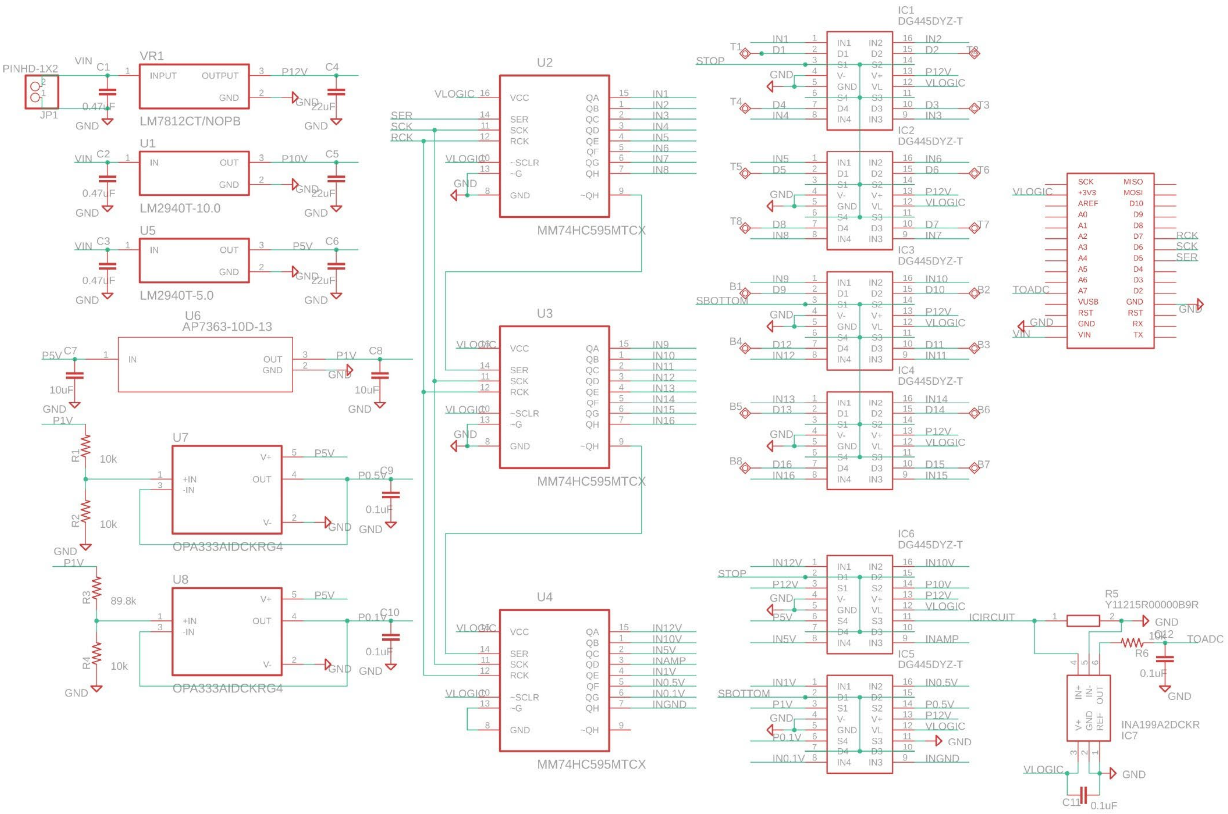

Supplementary Figure 28. Circuit diagram for 8x8 memristor electronic system

Supplementary Notes

Supplementary Note 1 Explanation for the transient increase in the current decrease during the process of negative charge on the Al electrode in a weakly alkaline buffer solution of pH=10.

In a weakly alkaline buffer environment of pH=10, when a positive voltage is applied to the Al electrode, the Al surface will be corroded to form Al $(OH)_3$ precipitation. Then, if we apply a negative voltage to the Al electrode (reverse voltage), bubbles and local hydroxide ions will be generated on the Al surface due to the electrolysis of water. In this case, the high concentration of hydroxide ions on the surface of the Al electrode will increase the local pH of the Al electrode. According to the phase diagram [1], [2] of Fig. S1, this will promote the transformation of Al $(OH)_3$ to AlO_2^{2-} ions, that is, the Al $(OH)_3$ precipitation covering the surface of the Al electrode will be gradually dissolved. As shown in Fig.1D, when a negative voltage is first applied to the Al electrode, the surface current gradually decreases. During this period, the current slowly increased, which is due to the local high pH promoting the dissolution of Al $(OH)_3$ into AlO_2^{2-}, resulting in more new Al surfaces and reaction sites.

Supplementary Note 2 Preferences for diodes and memristor devices under acidic/alkaline conditions

Diode preference under acidic conditions (pH=4): Due to the dense and thermodynamically stable oxide layer produced by anodic oxidation in acidic environments (negligible dissolution rate), the oxide layer exhibits irreversible unidirectional thickening with increasing operating time or continuous voltage. While this physical process causes initial memristor behavior drift, it provides a solid structural foundation for long-term stable diode rectification characteristics. Therefore, Figure 3F shows long-term rectification testing at pH=4 to demonstrate its application potential as a stable diode. **Memristor preference under alkaline conditions (pH=10):** In alkaline environments, the generated $Al(OH)_3$ products have a loose structure and are in a dynamic equilibrium of "easy formation - easy dissolution." This instability, however, endows the device with excellent write/erase capacity. The oxide layer can be thinned or removed by reverse biasing, thus avoiding unidirectional drift of characteristics. Therefore, an alkaline environment is indeed an ideal medium for achieving high-performance, high-cycle-frequency memristor behavior.

Supplementary Note 3 Formation of the hydrogel-electrode interface.

In the process of preparing the fabric-based 6*6 array, we first designed carbon and aluminum-based yarns on the upper and lower surfaces of cotton textiles respectively through a sewing process. Then, hydrogel was deposited on the textile by spraying a hydrogel solution. Since cotton textile has good hydrophilicity by nature, gelatin-based hydrogel contains a large number of hydrophilic functional groups such as hydroxyl and carboxyl groups. Therefore, the gelatin-based hydrogel solution can quickly infiltrate the fabric surface and penetrate into the pores of the fabric under the action of capillary effect [3], [4]. This process based on spraying hydrogel can make it infiltrate and diffuse on the surface of the electrode array and quickly form a uniform electrolyte layer.

Supplementary Note 4 Design of *microcontroller for multi-channel signal acquisition*

(1) System Architecture (Fig. R16): We developed an 8×8 switch matrix controlled by a microcontroller (MCU) via cascaded shift registers. This setup enables precise addressing of any individual memristor at the (T_j, B_i) intersection while keeping unselected lines in a high-impedance state to minimize crosstalk.

(2) Integrated Operations: Write Mode: The system routes regulated programming voltages (5V, 10V, or 12V) to the selected column while clamping the selected row to GND. **Read Mode:** To avoid negative supply rails, the polarity is reversed. A read bias (0.1V, 0.5V, or 1.0V) is applied to the row, and the resulting current is routed from the column to a sensing network.

(3) Signal Acquisition and Processing: The sensing circuit utilizes a precision shunt resistor and a differential amplifier (G=100). Signals are filtered and digitized by the MCU's onboard ADC. To ensure accuracy, each data point is an average of 20 ADC samples, effectively reducing random noise.

(4) Portability and Validation: The entire system operates from a single 14 V supply, with all bias levels generated internally via regulators and buffers. The accuracy of the readout chain was validated using a programmable decade box, ensuring reliable resistance characterization in a wearable form factor.

Supplementary Tables

Compared to hole/electron heterojunction diodes, these ion-based diodes do not show an advantage in response time. However, they show significant advantages in biocompatibility, flexibility, stretchability, and manufacturing complexity, thus possessing potential applications in implantable electronics, electronic skin, and fabric electronics, such as low-frequency rectification and Boolean logic gates (AND Gate, OR Gate). We also provide rectification data and response times for ion-based diodes from other studies, as shown in Table S1.

Supplementary Table 1 Comparison of ionic-based diode device on hysteresis time

	Ionic current rectification	Hysteresis time
This work	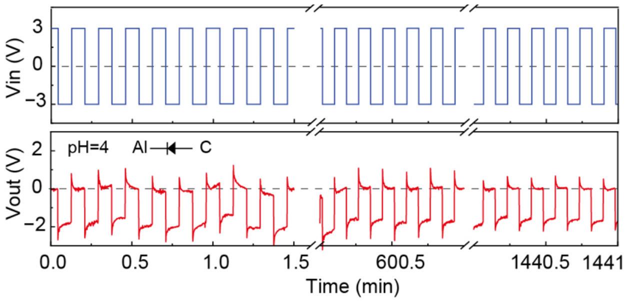	~5 s
[1] ***Science*** 2024, 386, 1024–1030	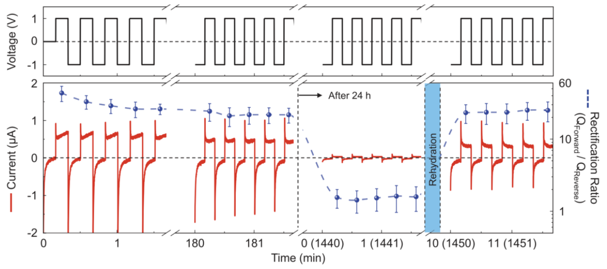	~10 s
[2] ***Adv. Funct. Mater.*** 2012, 22, 625–631	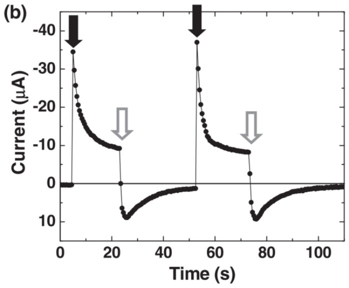	~20 s
[3] ***Science*** 367, 773–776 (2020)	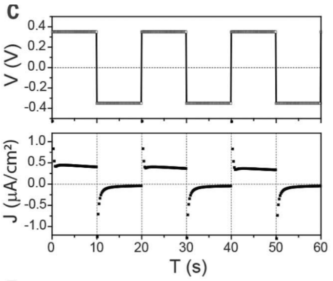	~10 s

Supplementary References

[1] Leung, Oi Man, et al. "Progress in electrolytes for rechargeable aluminium batteries." *Journal of The Electrochemical Society* 168.5 (2021): 056509.

[2] Tu, Jiguo, et al. "Nonaqueous rechargeable aluminum batteries: progresses, challenges, and perspectives." *Chemical reviews* 121.8 (2021): 4903-4961.

[3] Wang, Xianfeng, et al. "Biomimetic fibrous murray membranes with ultrafast water transport and evaporation for smart moisture-wicking fabrics." *ACS Nano* 13.2 (2018): 1060-1070.

[4] McCulloh, Katherine A., John S. Sperry, and Frederick R. Adler. "Water transport in plants obeys Murray's law." *Nature* 421.6926 (2003): 939-942.
