# Supplementary material for: In-fibre logic and memory via tuneable passivation–corrosion
Source: Nat Commun. 2026 Mar 31;17:4666. doi: 10.1038/s41467-026-71249-7 (PMC13201798; doi:10.1038/s41467-026-71249-7)
Supplement: Supplementary file 2 — Description of Additional Supplementary Information [file 41467_2026_71249_MOESM2_ESM.pdf]

Description of Additional Supplementary Files

File Name: Supplementary Movie 1
Description: FLAME is integrated into textiles by weaving it as a functional yarn.

File Name: Supplementary Movie 2
Description: Flame diode’s voltage-current characteristics.

File Name: Supplementary Movie 3
Description: Electrical signal response of memristor under continuous stimulation (Acidic conditions)

File Name: Supplementary Movie 4
Description: Electrical signal response of memristor under continuous stimulation (Alkaline conditions)

File Name: Supplementary Movie 5
Description: Textile memristor array preparation procedure.
